# Women have higher levels of CoQ_10_ than men when supplemented with a single dose of CoQ_10_ with monoglycerides omega-3 or rice oil and followed for 48 h: a crossover randomised triple blind controlled study

**DOI:** 10.1017/jns.2021.106

**Published:** 2022-01-18

**Authors:** Sandrine Beaulieu, Annick Vachon, Mélanie Plourde

**Affiliations:** 1 Centre de Recherche sur le Vieillissement, Centre Intégré Universitaire de Santé et Services Sociaux de l'Estrie – Centre Hospitalier Universitaire de Sherbrooke, Sherbrooke, Québec, Canada; 2 Faculté de Médecine et des Sciences de la Santé, Université de Sherbrooke, Sherbrooke, Québec, Canada

**Keywords:** CoQ10, Crossover study, Monoglyceride omega-3, Pharmacokinetics, AUC, area under the curve, *C* 0 h, plasma concentration at baseline, *C* 48 h, plasma concentration at 48 h post-single-dose intake, *C*_max_, maximum concentration, CoQ_10_, coenzyme Q_10_, CoQ_9_, coenzyme Q_9_, MAG, monoglyceride, OM3, omega-3 fatty acids, *T*_max_, time to reach maximum concentration

## Abstract

Coenzyme Q_10_ (CoQ_10_), a lipid involved in ATP synthesis, exhibits very limited oral absorption, and its endogenous production decreases with ageing and with the occurrence of oxidative stress. Our group previously showed that monoglycerides omega-3 (MAG-OM3) increase OM3 plasma concentrations. Since CoQ_10_ is liposoluble, we hypothesised that its 48 h pharmacokinetics is higher when provided with MAG-OM3 compared to CoQ_10_ alone (in powder form) or added to rice oil (a neutral triacylglycerol oil). A randomised triple-blind crossover study was performed with fifteen men and fifteen women consuming the three supplements providing 200 mg of CoQ_10_ in a random order. Blood samples were collected before (*t* = 0) and 1, 3, 5, 6, 7, 8, 10, 11, 24 and 48 h after the supplement intake. Plasma total CoQ_10_ concentrations were analysed on ultrahigh-performance liquid chromatography coupled to a tandem mass spectrometer (UPLC-MS/MS). Participants were 26⋅1 ± 4⋅8 years old. When CoQ_10_ was provided with rice or MAG-OM3 oils, the 48 h area under the curve (AUC 0–48 h) was approximately two times higher compared to when provided without an oil. The delta max concentration (Δ*C*_max_) of plasma CoQ_10_ was, respectively, 2 (MAG-OM3) and 2⋅5 (rice oil) times higher compared to CoQ_10_ alone. There was a significant sex by treatment interaction (*P* = 0⋅0250) for the AUC 0–6 h supporting that in postprandial, men and women do not respond the same way to the different supplement. Women had a higher CoQ_10_ concentration 48 h after the single-dose intake compared to men. We conclude that CoQ_10_ supplements must be provided with lipids, and their kinetics is different between men and women.

## Introduction

Coenzyme Q_10_ (CoQ_10_), also known as ubiquinone, is an endogenously synthesised fat-soluble molecule. It is composed of a benzoquinone ring and 10 isoprene subunits^(1)^. It has two main roles in the body. CoQ_10_ plays a major role in ATP synthesis by transporting electrons from complexes I and II to complex III via the Q cycle in the electron transport chain of the mitochondria and it also contributes to the proton gradient^(2,3)^. CoQ_10_ is also a scavenger of free radicals, can regenerate other antioxidants’ pools like vitamin E^(4)^ and can protect lipoproteins from lipid peroxidation at the initiation and propagation stages^(2)^. A low level of blood CoQ_10_ has been reported in those with chronic diseases such as cardiovascular diseases, neurodegenerative diseases, diabetes and cancer^(5,6)^. Moreover, during ageing, CoQ_10_'s blood levels tend to decrease^(7,8)^.

The total amount of CoQ_10_ in the body is approximately 2000 mg. Its daily nutritional intake is estimated at 3–6 mg/day but the body needs are estimated to be ~500 mg of CoQ_10_ every day^(9)^. Therefore, the consumption of 30–100 mg of CoQ_10_ per day by healthy people and of 60–1200 mg of CoQ10 per day in those with a medical condition as described above is recommended^(9)^. CoQ_10_ supplementation is considered safe and well tolerated without any serious adverse effects in human subjects^(10)^. Its acceptable daily intake is 12 mg/kg/d which is about 720 mg/d for a person weighing 60 kg^(10)^. Some studies even used CoQ_10_ doses up to 2400 mg/d, which was well tolerated with no safety concerns reported^(11)^. Furthermore, it seems that dietary CoQ_10_ intake does not influence its endogenous synthesis and there is no long-term accumulation in the tissues^(10)^. The main issue with CoQ_10_ supplementation is its low and incomplete absorption, and hence, its efficacy when provided as a supplement is doubted or limited^(12)^. To overcome this issue, the most popular approaches focused on adding synthetic products such as liposomes, lipid-free nano-formulations, water-soluble CoQ_10_, CoQ_10_-loaded oleo gels and CoQ_10_ micellisation. Although these strategies have improved CoQ_10_ absorption and bioavailability, these synthetic products have no health benefits^(12)^.

Our group previously conducted pharmacokinetic studies on omega-3 fatty acids (OM3) esterified in monoacylglycerols (MAG) and reported that plasma OM3 concentrations were two to three times higher 0–6 h post dose intake when OM3s were esterified in MAGs compared to when esterified in ethyl esters or triacylglycerols^(13,14)^. The amphipathic nature of MAGs contributes to improving the emulsification of fats and producing micelles in the gastrointestinal tract^(15–17)^. Therefore, we hypothesise that CoQ_10_ blood levels over a 48 h follow-up will be higher when provided with MAG-OM3 than CoQ_10_ alone (in powder form) or added to rice oil (a neutral triacylglycerol oil). The objective of the present study was to perform, in men and women, comparative pharmacokinetics of the three CoQ_10_ formulations and monitor concentrations of total CoQ_10_ in the plasma over 48 h after the single-dose intake.

## Methods

### Study participants

The present study was a randomised, triple-blind, crossover trial conducted at the Research Center on Aging, Centre Intégré Universitaire de Santé et des Services Sociaux de l'Estrie – Centre Hospitalier Universitaire de Sherbrooke (CIUSSSE–CHUS), in Sherbrooke (Quebec, Canada). This trial was revised and approved by the Research Ethics Committee of the CIUSSSE–CHUS (reference no. 2020-3280). The present study is registered at clinicaltrials.gov under no. NCT04035525.

Recruitment took place from June to December 2020. The main inclusion criteria were to be aged between 18 and 50 years old. Exclusion criteria included people with a particular diet such as being vegetarian or vegan, and/or with allergies to fish/seafood, people taking CoQ_10_ and/or omega-3 fatty acid supplements or who had taken these supplements daily in the previous 6 months. People with a body mass index of <18⋅5 kg/m^2^ or >34⋅9 kg/m^2^ were also excluded and those smoking tobacco or marijuana as well. Other exclusion criteria assessed during the phone call were: current or past performance athletes; alcohol and/or drug abuse; malnutrition; diabetes; presence of systemic, gastrointestinal, hepatic, renal, cardiac, thyroid or hormonal problems; or a diagnosis of schizophrenia, psychotic disorder, bipolarity, major depression (<5 years), panic disorder and/or obsessive-compulsive disorder. Also, women who were pregnant or lactating or going through menopause and people who had donated blood in the past month were excluded. Females of childbearing age were required to use a contraceptive method to avoid getting pregnant during the trial. After phone screening, participants were invited to the Research Center. At that time, participants were requested to read and sign the informed consent form. All participants provided written informed consent prior to the trial beginning. The fasted blood samples were then collected to assess blood biochemistry at baseline. These analyses were performed at the Centre Hospitalier Universitaire de Sherbrooke clinical laboratory and included HDL cholesterol, LDL cholesterol, triacylglycerols, fasting blood glucose concentration and glycated haemoglobin. Individuals with values outside the clinical reference range were excluded.

### Randomisation and blinding

**Fig. 1.**
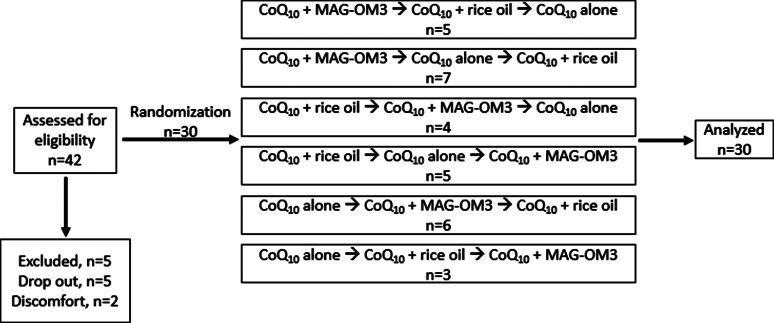
Clinical trial flow chart and randomisation of the treatments order to the CoQ_10_ + MAG-OM3, CoQ_10_ + rice oil or CoQ_10_ alone supplement. The *n* represents the number of participants allocated to the treatment sequence. CoQ_10_, coenzyme Q_10_; MAG-OM3, monoglycerides omega-3.

Randomisation of the treatment groups was performed using ‘https://www.randomizer.org/’. Treatment randomisation is described in a flow chart in Fig. 1. Participants, clinical staff and laboratory staff were blinded to treatment allocation. The CoQ_10_ + MAG-OM3 and CoQ_10_ + rice oil capsules were identical in size, shape and taste, but differed from the CoQ_10_ alone capsule. To maintain blinding, participants wore an eye mask when taking the supplements and a research staff not involved in the trial provided the capsules to participants to keep blinding. Also, each plasma sample was assigned a random number between 1 and 990 to avoid knowing the participant's number, treatment and the time at which the sample was analysed. These were unblinded only after performing all the CoQ_10_ analyses described below.

### Study treatments

The study treatments consisted of (1) CoQ_10_ + MAG-OM3, (2) CoQ_10_ + rice oil (a neutral triacylglycerol oil) and (3) CoQ_10_ alone, in powder. Each capsule provided 100 mg of oxidised CoQ10, the most stable form of CoQ_10_, and 500 mg of oil, except for the CoQ_10_ alone which contained only 100 mg of CoQ_10_ in powder. The supplement content in CoQ_10_ was analysed by Labs Mart and it ranges from 108 to 117 mg/capsules (*n* 2 capsules/supplement type). The fatty acid profile of the capsules was determined using the modified AOCS Ce 1b-89 method and is detailed in Table 1.

**Table 1:** Fatty acids content in the supplements

	CoQ_10_ + MAG-OM3 supplement (mg/capsule)		CoQ_10_ + rice oil supplement (mg/capsule)	
Fatty acids	Mean	SD	Mean	SD
8:0	0·0	0·0	0·4	0·5
10:0	0·9	0·4	3·4	3·7
14:0	1·5	0·0	1·5	0·1
16:0	6·1	0·7	89·6	2·8
16:1 n-7	0·5	0·0	0·9	0·0
17:1	0·4	0·0	0·1	0·3
18:0	6·6	0·0	9·5	0·3
18:1 n-9	11·3	0·8	190·4	5·8
18:1 n-7	3·3	0·0	4·0	0·1
18:2 n-6	3·6	0·7	144·1	4·4
18:3 n-6	0·7	0·0	1·6	0·1
18:3 n-3	1·2	0·0	4·3	0·1
20:0	5·2	0·1	3·6	0·1
20:1	14·3	0·2	2·4	0·1
20:2	2·4	0·1	0·0	0·0
20:3 n-6	2·9	0·0	0·0	0·0
20:4 n-6	12·9	0·2	0·0	0·0
20:3 n-3	1·3	0·1	0·0	0·0
20:5 n-3	224·3	3·5	0·0	0·0
22:0	0·0	0·0	1·3	0·1
22:1	1·7	0·1	0·0	0·0
22:5 n-6	3·4	0·1	0·0	0·0
22:5 n-3	14·3	0·1	0·0	0·0
22:6 n-3	95·3	1·6	0·0	0·0
24:0	0·0	0·0	2·5	0·1
**TOTAL Concentration**	414·1	8·9	459·8	18·7

n = 3 capsules/supplement form.

Participants consumed two capsules providing 200 mg of CoQ_10_ and 1 g of oil. This dose was selected because a study compared the pharmacokinetics of a single oral dose of 100 and 200 mg of CoQ_10_ and they reported an increase of 150 % of CoQ_10_ in the plasma with the 200 mg dose compared to an increase of 80 % with the 100 mg dose^(18)^. Another study also obtained similar results^(19)^. Therefore, the dose of 200 mg/d of CoQ_10_ was selected for the present study. Also, a dose of 1 g of oil was selected because it was similar to a previous study done by our group^(13,14)^. A minimum of 1 week washout period was mandatory between treatments since CoQ_10_'s half-life is 33 h^(20)^. It is generally accepted for drugs that four to five times the half-life eliminate the active molecule at 94–97 %, which is considered to be below clinical significance^(21)^. Therefore, in this trial, 6⋅9 d were required to eliminate CoQ_10_ at 97 %.

### Study design

Participants were required to be fasted at arrival. A blood sample was then collected, and a standardised breakfast with the two capsules was provided. The breakfast provided 410 kcal (75 % carbohydrates, 22 % proteins, 3 % lipids) and was composed of two toasts with raspberry jam, a banana, a protein chocolate milk and a coffee or a tea. In the present study, meals were provided throughout the day to control the dietary fat intake. A low-fat menu was selected to limit bias in the CoQ_10_ metabolism. After consuming the capsules, the timer indicated the subsequent blood samples to be collected at 1, 3, 5, 6, 7, 8, 10, 11, 24 and 48 h post-single oral dose intake. Blood was collected in tubes containing citrate. Lunch and dinner were provided at 4 and 9 h post-single-dose intake, and they provided, respectively, 614 kcal (78 % carbohydrates, 17 % proteins, 5 % lipids) and 696 kcal (72 % carbohydrates, 23 % proteins, 5 % lipids). Lunch was composed of one serving of chow mein, baby carrots, an apple, a yogurt, a juice and a fig bar, and dinner was composed of one serving of General Tao, one slice of bread, one mini cucumber, apple sauce, protein chocolate milk, vegetable juice and a granola bar. Also, at 11 h post-dose intake, a snack (one granola bar and one package of gummy fruits) provided 210 kcal (87 % carbohydrates, 6 % proteins, 7 % lipids). The menu and food items consumed by a participant in the first pharmacokinetic study day were kept the same for the other two pharmacokinetic days to limit potential bias. Participants returned for a blood sample 24 and 48 h post-single-dose intake. During the study day, blood samples were kept on ice and centrifuged at 1700 ***g*** for 10 min at 4°C, and the plasma was aliquoted and stored at −80°C until further analysis.

### CoQ_10_ extraction and analysis

Total CoQ_10_ was extracted from plasma samples, using the Hansen *et al.* method^(22)^. Because reduced CoQ_10_ is unstable, plasma CoQ_10_ was oxidised with 1,4-benzoquinone to generate a stable CoQ_10_ analyte. To allow quantification, coenzyme Q_9_ (CoQ_9_) was added as an internal standard.

Briefly, samples were thawed at room temperature for 45 min. In total, 50 μl of plasma were transferred to a 1⋅5 ml tube along with 50 μl of 1,4-benzoquinone dissolved in methanol (0⋅4 mg/ml) and 50 μl of CoQ_9_ dissolved in 1-propanol (800 μg/l). After mixing for 15 min at room temperature, 1 ml of 1-propanol was added to the tube. Tubes were then mixed again for 1 min and centrifuged at 10 000 ***g*** for 3⋅5 min at 4°C. After centrifugation, 1 ml of the supernatant was transferred to an HPLC vial. In total, 300 μl of methanol was added to have the same solvent as the mobile phase of the liquid chromatography system. All vials were stabilised in the autosampler at 4°C for 30 min before being injected. One quality control sample was done per batch.

Analyses were performed on Waters Acquity UPLC H-Class system coupled to Waters Xevo TQ-S Micro tandem mass spectrometer. The column used was Waters Acquity UPLC BEH C18 2⋅1 × 50 mm, 1⋅7 μm and the pre-column was Waters Acquity UPLC BEH C18 Vanguard 2⋅1 × 5 mm, 1⋅7 μm and it was maintained at 25°C. The mobile phase was composed of methanol with 5 mm of ammonium formate, in isocratic mode. The injection volume was 1 μl, the flow was 0⋅4 ml/min and the total run time was 14 min per injection. The mass spectrometer was operated in the electrospray positive mode. The optimised detector parameters were set as follows: nitrogen desolvation was at 600°C with a flow rate of 1000 l/h, the nitrogen cone gas was set at 80 l/h, the argon collision gas was at 5 psi, the source temperature was as 150°C and the capillary voltage was set at 0⋅9 kV. The cone voltage was at 88 V for CoQ_10_ and 82 V for CoQ_9_. The collision energy was at 32 V for CoQ_10_ and at 30 V for CoQ_9_. Quantification was performed in multiple reaction monitoring mode with the following mass transitions: CoQ_10_ at m/z 863⋅6 → 197⋅1 and CoQ_9_ at 795⋅6 → 197⋅1. The dwell time for each transition was 0⋅037 s.

Calibration curves were freshly prepared daily using CoQ_10_ stock solution at the following concentrations: 0, 250, 500, 1500, 2000 and 2500 μg/l. An internal standard (CoQ_9_) at a concentration of 800 μg/l was also added in each of the calibration curve samples. Calibration curves were generated using MassLynx software by plotting the peak area ratio of CoQ_10_ to internal standard *v.* the CoQ_10_ concentration. Sample CoQ_10_ concentrations were calculated with a linear regression using the 1/*x* weighting factor.

### Statistical analysis

Sample size was calculated with a website sample size calculator^(23)^. The sample size was calculated based on the available data on the pharmacokinetics of CoQ_10_ that was the closest to our study design. López-Lluch *et al.* performed a pharmacokinetic study on seven different CoQ_10_ formulations and monitored CoQ_10_ plasma concentration over a 48 h period in fourteen healthy adults after a single oral dose intake of approximately 100 mg of CoQ_10_^(24)^. Using the 0–48 h AUC of the olive oil/coconut oil + CoQ_10_ treatment (6⋅28 ± 3⋅07 mg/l*48 h) compared to the finely ground CoQ_10_ powder (8⋅94 ± 3⋅33 mg/l*48 h), we calculated, with a power of 80 % with an *α* = 0⋅05, a sample size of twenty-five participants was required. We, therefore, tested thirty participants to account for a potential 20 % attrition.

Area under the curves (AUC) were calculated using GraphPad Prism 7.03 software. The incremental AUC was calculated with the 48 h-curves, by ignoring the areas below the baseline. Δ*C*_max_ was defined as the CoQ_10_ maximum concentration minus the baseline CoQ_10_ plasma concentration. *T*_max_ was defined as the time required to reach *C*_max_.

The primary outcome of the present study was the 0–48 h AUC of CoQ_10_. Secondary outcomes included 0–6 h AUC, Δ*C*_max_, *T*_max_, *C* 0 h and *C* 48 h. Statistical analyses were performed using IBM SPSS Statistics 25 and GraphPad Prism 7.03 software. For the pharmacokinetic parameters, the Shapiro–Wilk normality test (*α* = 0⋅05) was performed, and data were not normally distributed. Therefore, Friedman's ANOVA statistical rank test for paired samples was used. Dunn's multiple comparison test was used for the comparison between treatments. For the comparison between sexes, a two-way ANOVA test was performed, with Bonferroni's multiple comparison test. However, for the Δ*C* 48 h, these data were analysed by fitting a mixed model, rather than by repeated-measures ANOVA, because there were missing values. Indeed, three participants refused to come back on one of their 48 h follow-up visits. The *P* interaction represents the combined effect of sex and supplement type, while *P* sex and *P* supplement represent the individual effects of sex or supplement.

## Results

### Participants baseline characteristics

Table 2 presents the baseline data of the participants. There was no significant difference between men and women. The participants’ mean age was 26⋅1 ± 4⋅8 years. Treatment randomisation is described in the flow chart (Fig. 1).

**Table 2:** Baseline characteristics of the participants

	Total cohort (n = 30)		Men (n = 15)		Women (n = 15)		
Baseline characteristics	Mean	SD	Mean	SD	Mean	SD	P-value
Age (years)	26·1	4·8	26·7	4·3	25·5	5·2	0·4771
BMI (kg/m^2^)	25·3	3·6	25·6	3·5	25·1	3·8	0·7043
Plasma TG* (mmol/L)	0·9	0·6	0·9	0·4	1·0	0·7	0·9584
Plasma HDL-C (mmol/L)	1·4	0·3	1·3	0·3	1·4	0·2	0·1738
Plasma LDL-C (mmol/L)	2·5	0·8	2·5	0·8	2·4	0·8	0·7934
Plasma glucose (mmol/L)	4·5	0·4	4·4	0·4	4·5	0·4	0·5183
HbA1c (%)	5·0	0·3	5·1	0·3	5·0	0·3	0·6834

BMI = body mass index, TG = triglycerides, HDL-C = high-density lipoproteins cholesterol, LDL-C = low-density lipoproteins cholesterol, HbA1c = glycated hemoglobin. P values were evaluated by T test for unpaired measurements.

*For TG, the data was not normally distributed, and a nonparametric Mann-Whitney test was performed.

### Primary outcome

**Fig. 2.**
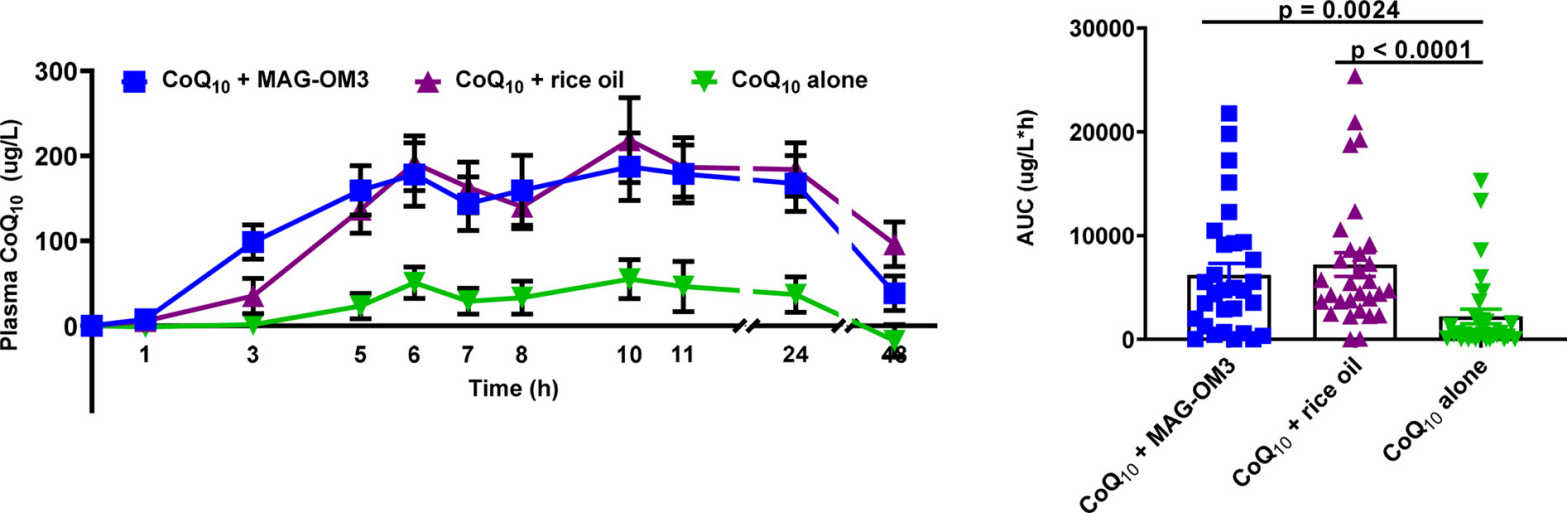
Plasma CoQ_10_ concentrations over a 48 h period when combined with MAG-OM3, rice oil or alone. The results are expressed as the mean ± sem. *P* values were assessed by the Friedman nonparametric statistical analysis. CoQ_10_, coenzyme Q_10_; MAG-OM3, monoglycerides omega-3; AUC, area under the curve.

Fig. 2 and Table 3 present the 0–48 h pharmacokinetic curves and pharmacokinetic parameters of CoQ_10_. The 0–48 h AUC of CoQ_10_ combined with MAG-OM3 or rice oil was significantly higher than CoQ_10_ provided alone (Table 3).

**Table 3:** Pharmacokinetic parameters of CoQ_10_ in combination with MAG-OM3, rice oil or alone.

	CoQ_10_ + MAG-OM3		CoQ_10_ + Rice oil		CoQ_10_ alone		
Pharmacokinetic parameters	Mean	SEM	Mean	SEM	Mean	SEM	P-value
Tmax (h)	9·5	1·3	9·6	1·3	6·9	1·1	0·6128
C0h (μg/L)	611·6	42·0	617·9	43·4	581·8	40·2	0·0877
ΔCmax (μg/L)	281·1 ^A^	44·5	325·1 ^A^	50·1	127·8 ^B^	26·5	0·0015
ΔC48h (μg/L)	38·3 ^A^	20·5	96·1 ^B^	26·3	−17·7 ^A^	19·2	0·0004
AUC 0-6h (μg/L*h)	576·7 ^A^	100·0	456·1 ^A^	73·1	161·7 ^B^	41·6	<0·0001
AUC 0-48h (μg/L*h)	6211·0 ^A^	1097·0	7216·0 ^A^	1148·0	2199·0 ^B^	702·6	<0·0001

Results are expressed as mean ± SEM. Tmax = time at which the maximum concentration is reached, C0h = plasma concentration at baseline, ΔCmax = maximum concentration, C48h = plasma concentration 48h post single dose intake, AUC 0-6 h = area under the curve over 6h, AUC 0-48h = area under the curve over 48h. P values were assessed by Friedman nonparametric statistical analysis, with Dunn's multiple comparison test. Data with different letters A and B are statistically different.

### Secondary outcomes

The 0–6 h AUC of CoQ_10_ was significantly higher when CoQ_10_ was combined with MAG-OM3 or rice oil compared to CoQ_10_ alone (*P* < 0⋅0001). Plasma CoQ_10_ peaked 6 h and again 10 h post-single-dose intake (Fig. 2). *T*_max_ and CoQ_10_ plasma concentrations at baseline were not different between treatments. CoQ_10_ concentrations still in the plasma 48 h after taking the supplements were 1⋅5–4⋅5 times higher for the CoQ_10_ + rice oil supplement compared to MAG-OM3 or CoQ_10_ alone (*P* = 0⋅0004) (Table 3). The Δ*C*_max_ of plasma CoQ_10_ was, respectively, 1 and 1⋅5 times higher when CoQ_10_ was provided with MAG-OM3 or rice oil compared to the powder supplement form (*P* = 0⋅0015) (Table 3).

**Fig. 3.**
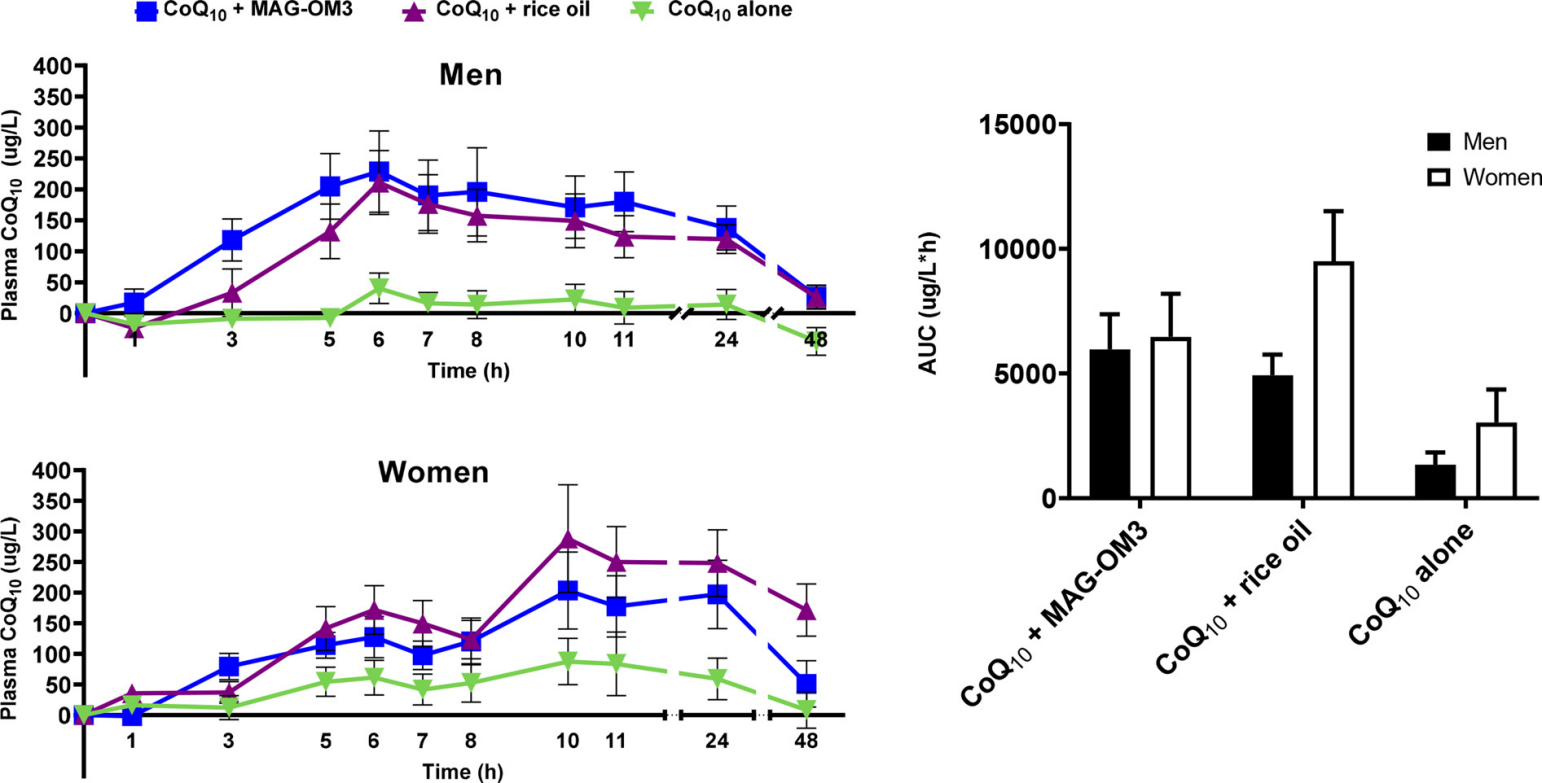
Plasma CoQ_10_ concentration in men and women over a 48 h period when combined with MAG-OM3, rice oil or alone and AUC over a 48 h period. The results are expressed as the mean ± se. CoQ_10_, coenzyme Q_10_; MAG-OM3, monoglycerides omega-3; AUC, area under the curve.

CoQ_10_ pharmacokinetic parameters were also assessed in relation to sexes (Fig. 3). There was no sex difference in the AUC 0–48 h, *T*_max_ and Δ*C*_max_. Forty-eight hours after the single-dose intake of CoQ_10_, women had, on average, eleven times higher CoQ_10_ plasma concentration than men (*P* = 0⋅0143). There was an interaction between sex and supplement form for the baseline concentration (*C* 0 h) and the AUC 0–6 h in the pharmacokinetics of CoQ_10_ between men and women (Fig. 3; Table 4). A significant sex by supplement interaction means that the effect of the supplements differs between men and women, even though the effect of sex alone is not significant. For the AUC 0–6 h, men had higher postprandial CoQ_10_ levels, whereas the highest concentrations in CoQ_10_ in women were reached 10 h after the single-dose intake.

**Table 4:** Pharmacokinetic parameters of CoQ_10_ in combination with MAG-OM3, rice oil or alone in men and women

Pharmacokinetic parameters	CoQ_10_ + MAG-OM3				CoQ_10_ + Rice oil				CoQ_10_ Alone			
	Men		Women		Men		Women		Men		Women		P-value		
	Mean	SEM	Mean	SEM	Mean	SEM	Mean	SEM	Mean	SEM	Mean	SEM	P interaction	P sex	P supplement
Tmax (h)	7·2	1·5	11·7	2·1	8·7	1·7	10·6	1·9	7·7	1·9	6·1	1·0	0·1772	0·3127	0·1795
C0h (μg/L)	589·6	54·6	633·6	65·3	658·7	58·4	577·1	64·4	614·8	64·5	548·9	48·9	0·0198	0·6691	0·2706
ΔCmax (μg/L)	279·8	67·6	282·4	60·2	373·1	52·1	373·2	85·8	87·0	18·4	168·7	48·3	0·5550	0·3588	0·0003
ΔC48h* (μg/L)	26·5	18·8	51·0	37·9	25·6	18·9	171·5	42·6	-45·5	22·2	8·3	30·0	0·0696	0·0143	0·0002
AUC 0-6h (μg/L*h)	731·3	177·2	422·0	81·3	452·2	113·5	460·1	96·3	99·9	35·3	223·4	73·4	0·0250	0·6176	<0·0001
AUC 0-48h (μg/L*h)	5957·2	1414·1	6464·9	1725·0	4927·9	824·8	9503·1	2007·1	1348·6	489·7	3049·5	1304·5	0·1707	0·1453	<0·0001

Tmax = time at which the maximum concentration is reached, C0h = plasma concentration at baseline, ΔCmax = maximum concentration, ΔC48h = plasma concentration 48h post single dose intake, AUC 0-6 h = area under the curve over 6h, AUC 0-48h = area under the curve over 48h. P values were assessed by a 2-way ANOVA statistical analysis, with Bonferroni's multiple comparison test. The P interaction represents the combined effect of sex and type of supplement while P sex and P supplement represent the individual effect of sex or supplement.

*For the ΔC48h, these data were analyzed by fitting a mixed model, rather than by repeated measures ANOVA because there were missing values, i.e. 3 participants refused to come back on one of their 48h follow-up visit (n = 3).

## Discussion

In the present study, we hypothesise that CoQ_10_ blood levels over a 48 h follow-up is higher when provided with MAG-OM3 than CoQ_10_ alone (in powder form) or added to rice oil (a neutral triacylglycerol oil). We report here that both CoQ_10_ + MAG-OM3 and CoQ_10_ + rice oil had approximately two times higher AUC 0–48 h than CoQ_10_ alone, and hence, our hypothesis is rejected. The rationale for using MAG-OM3 in the present study stem from a previous study done by our group supporting that when provided esterified in MAG, OM3 plasma concentrations were twice than when provided in a triacylglycerol or ethyl ester form and that concentration in the plasma 24 h after the single dose was also twice that of the other supplements^(14)^. Moreover, our previous study also showed that there is an early effect of MAG-OM3 on fat absorption, and this would improve the AUC 0–6 h of CoQ_10_ compared to other formulations. However, our data showed that AUC 0–6 h was higher when CoQ_10_ was provided with MAG-OM3 and rice oil compared to when provided alone. Therefore, it seems that providing CoQ_10_ with MAG-OM3 did not reproduce the same postprandial benefit observed in Chevalier *et al.*^(14)^ when investigating the metabolism of OM3. This suggests that liposoluble molecules like CoQ_10_ might only require to be solubilised in an oil to improve its absorption. However, its metabolism seems to be biphasic.

Indeed, we observed a biphasic shape of the CoQ_10_ pharmacokinetic curve with a CoQ_10_ peak at 6 h and again 10 h post-single-dose intake. This two-peak pattern has been reported in previous pharmacokinetic studies of CoQ_10_^(9,25–27)^. For instance, Evans *et al.*^(25)^ reported a biphasic distribution of CoQ_10_, with a first peak 6 h and a second peak between 12 and 24 h post-dose intake^(25)^. One main explanation for this biphasic distribution is related to CoQ_10_ redistribution from tissues and its enterohepatic recycling after food intake 4 and 9 h post-single-dose intake^(27,28)^. Lamber and Parks^(28)^ hypothesised that ‘lipids secreted at the very onset of a meal are those that were consumed in an earlier meal, suggesting the presence of an enterocyte storage pool for triglycerides’^(28)^. Thus, we hypothesise that CoQ_10_ might have been stocked in the liver or other tissues enriched in lipids and thereafter redistributed to the blood circulation after food intake^(9,29)^. However, our results offer another explanation for this biphasic shape of CoQ_10_ metabolism. Indeed, our data support a difference in the COQ_10_ metabolism between men and women since we found that CoQ_10_ concentrations peaked at 10 h post-dose in women but at 6 h post-dose in men (Fig. 3). Other evidence is related to a sex by supplement interaction for AUC 0–6 h and a trend towards this interaction for Δ*C* 48 h (*P* = 0⋅0696). These results support that the CoQ_10_ metabolism in response to a CoQ_10_ supplement differs between men and women. Hence, men had a higher AUC 0–6 h than women and their concentration remained higher up to 24 h after the single-dose intake but returned close to baseline 48 h after the single-dose intake. On the other hand, 24 h after the single CoQ_10_ dose with MAG-OM3 or rice oil, women had ~30–60 % higher CoQ_10_ plasma concentrations than men and 48 h after the single-dose intake, women still had at least twice the CoQ_10_ plasma concentration level of men. Unlike the data of Wahlqvist *et al.*^(30)^ suggesting that men have a better absorption and/or lower clearance of CoQ_10_ than women, here our data suggest that woman actually have lower clearance since their plasma CoQ_10_ concentrations are higher than men 24 and 48 h after the single-dose intake. This could be partially explained by a slower gastric motility^(31)^ in women supporting why CoQ_10_ absorption peaked later in women, although *T*_max_ was not statistically different between men and women. However, from our data, it seems that there are transiently higher concentrations of CoQ_10_ in women than men and there was a trend towards a sex by supplement interaction (*P* = 0⋅0696) in the Δ*C* 48 h concentration. Delta plasma CoQ_10_ concentrations in women receiving the CoQ10 + rice oil were about eleven times higher than the other supplements. Looking back at the data to see if this was the result of one or two outliers, we can confirm that it was not the case and that nine participants out of fifteen had a plasma concentration higher than 80 μg/l of CoQ_10_.

In the present study, we also found a sex by supplement interaction on the AUC 0–6 h. In the literature, there are discrepancies between studies in the AUC 0–6 h, and this result should therefore be interpreted with caution. For instance, one study reported a higher AUC^(32)^, while another reported a higher concentration^(30)^ in men than women after a single-dose intake of CoQ_10_. Conversely, one study reported higher CoQ_10_ bioavailability in women than in men, although this result was not significant^(26)^. Therefore, it is highly recommended that future pharmacokinetics of CoQ10 should be evaluated in men and women since their absorption peak is different by sex together with their levels up to 48 h after the single-dose intake. This recommendation also needs to consider the baseline CoQ_10_ concentrations since previous studies reported higher levels in men than women^(33–36)^. In the present study, there was an interaction between sex and supplement for *C* 0 h but our *post hoc* analysis was not able to determine where the differences were. We did not detect a significant *P* sex effect of *C* 0 h in the present study (Table 4).

The present study had strengths and limitations. In terms of strengths, the crossover design was robust enough to detect significant differences between treatments. Also, every participant was their own control, which aimed at limiting inter-individual variability. Indeed, CoQ_10_ supplementation is known to generate plasma response with high inter-individual variability^(15,35,37,38)^ and although we had a strong research design, inter-individual variability remained important in the present study. The high inter-individual variability found in other studies could be explained by age, gender, redox state and health disorders^(9,37)^. Another strength of the present study is the standardised procedures. For example, all the participant's dietary intake during the study days was controlled, limiting bias among the study days especially with respect to dietary fat intake. Treatment randomisation and the triple-blind design were also strengths since neither the participants nor the clinical staff were aware of which supplement order the participants were subjected to and all tubes were coded to avoid knowing the sampling time, participant number and supplement type the tube corresponded to. The present study also has limitations. Since this is a pharmacokinetics study of a single oral dose, it does not provide information regarding the efficacy nor the long-term effects of the CoQ_10_ supplements. Also, it does not represent the pharmacodynamics of the supplements. The study population was young men and women which perhaps does not represent the population that could benefit the most from this type of supplementation. It also would have been interesting to test the pharmacokinetics parameters of these supplements in older adults, people under statin therapy or people with fat malabsorption since these groups of people could benefit more from the CoQ_10_ supplementation^(2,9)^ and their pharmacokinetics are likely to differ from the ones we obtained in the present study. Knowing that CoQ_10_ absorption is enhanced in the presence of lipids^(15)^, the low-fat diet throughout the study day could have influenced the overall CoQ_10_'s absorption downwards. However, the purpose of our control supplement (CoQ_10_ in powder form) was to evaluate CoQ_10_ absorption when it was administered alone, with limited oil consumption. It would not have been possible to evaluate this effect if provided with a high-fat diet. This is a limitation in the transfer of these results to the general population since most people eat a diet that has higher fat levels than the ones we provided here.

To conclude, the purpose of this pharmacokinetic study was to evaluate whether the 48 h pharmacokinetics of CoQ_10_ was enhanced when provided with MAG-OM3. Our results do not support this hypothesis since CoQ_10_ AUC 0–48 h was similar to when CoQ_10_ was provided with rice oil. We conclude that the administration of CoQ_10_ in combination with lipid carriers helps enhance its absorption and overall metabolism. However, one important finding of the present study is the different pharmacokinetics of CoQ_10_ between men and women, with higher level of CoQ_10_ in women than men when provided with MAG-OM3 or rice oil. The plus value of the MAG-OM3 supplements resides in its OM3 content and pharmacokinetics providing higher OM3 levels in the blood in postprandial. Specifically, these results are of potential interest for those with fat malabsorption issues but require to be tested in this population before a definite conclusion can be drawn.
